# Factors predicting serum clozapine levels in Middle Eastern patients: an observational study

**DOI:** 10.1186/s12888-022-03910-6

**Published:** 2022-04-15

**Authors:** Ahmed Hassab Errasoul, Mohammed A. Alarabi

**Affiliations:** Department of Psychiatry, College of Medicine, King Saud University, PO Box 7805, Riyadh, 11472 Kingdom of Saudi Arabia

**Keywords:** Clozapine, Serum, Schizophrenia, Middle East, Psychosis

## Abstract

**Background:**

Despite its superiority over other drugs for psychosis, clozapine remains underused and is associated with many clinical challenges, including difficulties in predicting therapeutic serum levels (350-600 ng/mL). We found no large or recent study that investigated the determinants of serum clozapine levels in Middle Eastern patients. Therefore, we investigated the association between clozapine dose and serum level, and the clinical predictors of the clozapine serum level, in Middle Eastern patients.

**Methods:**

This cross-sectional study included 94 patients of Middle Eastern ethnicity who attended the Clozapine Clinic in King Saud University Medical City in Riyadh, Saudi Arabia. We used a single measure of the serum clozapine level, which was collected 12 h after the last oral dose of clozapine under steady-state conditions.

**Results:**

The average clozapine dose and serum level were 400 mg/daily and 705 ng/mL, respectively. The majority of patients (59.8%) had serum levels higher than 600 ng/mL. Clozapine dose and serum level were positively correlated (*r*_*s*_ [94] = 0.32, *p* = 0.002). We generated a predictive model of the serum clozapine level, which revealed that the daily dose, smoking status, use of fluvoxamine or lamotrigine, and body mass index (BMI) predicted 43.6% of the variance in the serum level (*p* < 0.001). Using this model, we calculated that patients with a BMI of 25 kg/m^2^ would require a clozapine dose between 50 to 275 mg/daily if they were non-smokers, and a dose of 200 to 450 mg/daily if they were smokers, in order to reach a serum clozapine level between 350 to 600 ng/mL. Patients with higher BMI and those receiving fluvoxamine would require lower doses.

**Conclusions:**

This was a naturalistic study of the clozapine dose-level relationship and the clinical predictors of the serum clozapine level in a sample of Middle Eastern patients. The ratios of clozapine level to dose in our patients more closely resembled those reported in Asian samples than in European samples. These findings do not reduce the value of individualised therapeutic drug monitoring, but may assist clinicians when prescribing clozapine to Middle Eastern patients. Further psychopharmacological studies are needed on this demographic population.

**Supplementary Information:**

The online version contains supplementary material available at 10.1186/s12888-022-03910-6.

## Background

Clozapine has been demonstrated to be superior to other drugs for the treatment of schizophrenia symptoms [1–3]. It is the only approved medication for treatment-resistant schizophrenia [4]. However, clozapine remains underused in clinical practice [5], including in Saudi Arabia [6]. Furthermore, discontinuation of clozapine is common owing to nonadherence to monitoring protocols or drug-related side effects [7, 8]. Such clinical challenges may be the reason behind the underutilisation of clozapine in Saudi Arabia and other Gulf countries [9]. Increasing clinicians’ knowledge about clozapine dosing and the issues that might arise during treatment may help in overcoming the barriers to clozapine use [10]. One method of addressing some clinical concerns during clozapine treatment is therapeutic drug monitoring (TDM), which is the practice of measuring drug levels in the patient’s blood to adjust prescribed doses [11]. Measuring serum clozapine levels can help in dose titration, detecting toxicity, and ensuring adherence [12]. Clozapine is hepatically metabolized by the cytochrome P450 (CYP) family of enzymes, and primarily by the enzyme CYP1A2 [13]. In addition to the serum level of clozapine, the level of its major metabolite, N-desmethylclozapine, also known as norclozapine, is often measured during TDM [14]. Calculating the ratio of clozapine to norclozapine level (CLZ/NDMC ratio) can be clinically useful as well. A ratio lower than 0.5 indicates fast clozapine metabolism or recent nonadherence to medication. On the other hand, a ratio higher than 3 indicates inhibition of the metabolizing enzyme or enzymatic saturation due to high oral doses of the drug [15]. Many exogenous agents can influence CYP1A2 activity, and therefore affect clozapine metabolism, including tobacco smoke, which increases its activity; caffeine and oral contraceptives, which weakly or moderately inhibit its activity; and ciprofloxacin and fluvoxamine, which potently inhibit its activity [13].

Studies have traditionally concluded that a serum clozapine level of 350 ng/mL, or a level that falls between 250 and 550 ng/mL, is associated with clinical response [16–21]. Evidence suggests that serum levels exceeding 600–700 ng/mL are associated with lower rates of clinical responses and higher rates of dose-dependent side effects [22]. This translates to clozapine having a narrow therapeutic index of 350-600 ng/mL [23], which adds further import to careful dosing and drug level monitoring. Although the relationship between the drug level and clinical response is not always consistent, guidelines have nevertheless concluded that if a patient on clozapine does not demonstrate an adequate response, and the drug level is below 350 ng/mL, then the dose should be increased to achieve a level above 350 ng/mL [24].

Remarkably, serum clozapine levels exhibit significant inter-individual variability [25], which presents clinicians with the challenge of how to predict the therapeutic drug level if clozapine TDM is not available. The variability in serum clozapine levels may be due to various known factors that influence clozapine metabolism. These factors include female sex, cessation of cigarette smoking, obesity, systemic inflammation, and the concomitant use of other medications, all of which can lead to an increase in clozapine levels [26–31]. The variability in clozapine levels may also be related to ethnic differences in clozapine metabolism, with the factors of sex and smoking still being relevant within each ethnic group. For instance, East Asians have been observed to require almost half the clozapine dose to achieve the same serum levels as those in Europeans [32–34]. A recent international guideline defined personalized titration schedules for clozapine depending on ancestry [23]. The guideline estimated that the ratio of clozapine level (or concentration) to the daily dose of clozapine (known as the C/D ratio) in Asians and Native Americans ranges from 2.12 to 1.30 while the C/D ratio in Europeans ranges from 1.48 to 0.95. These findings illustrate how distinct relationships between clozapine level and dose in different ethnic groups can inform treatment. Moreover, findings from a study on 26 Middle Eastern patients from Saudi Arabia have suggested that Middle Eastern patients require doses lower than those required by European Caucasians to achieve the same therapeutic serum levels [18, 32, 35–37]. However, our literature review found no large or recent study that investigated the determinants of serum clozapine levels in Middle Eastern patients. Therefore, the main aims of this study were to explore the association between clozapine dose and serum level in an ethnically homogeneous group of Middle Eastern patients, address whether the relationship between clozapine dose and serum level in this population is closer to that found in Asians or in Europeans, and to identify which clinical factors other than daily clozapine dose are associated with the serum clozapine level. We hypothesised that the relationship between clozapine dose and serum level in our patient sample would be linear, as previously reported in other populations [18, 30, 35], and the most important predictors of variation in serum levels would be the daily dose of clozapine and the concomitant use of other psychotropic medications.

## Methods

This study was conducted at the Clozapine Clinic in King Khalid University Hospital (KKUH), part of King Saud University Medical City in Riyadh, Saudi Arabia. We included the data of all patients who attended the Clozapine Clinic in KKUH between December 2015 (the inception of the Clozapine Clinic in KKUH) and April 2020. We collected data from patients’ electronic medical records and laboratory records. The inclusion criteria were clozapine treatment and at least one measurement of the serum clozapine level. The exclusion criteria were undetectable serum clozapine level and missing data from electronic medical records regarding the clozapine dose or concomitant psychotropic drug use. Collected data included demographics, psychiatric diagnosis, body mass index (BMI), and concomitant use of psychotropic medications other than clozapine. All included patients were Saudi nationals of Middle Eastern ethnicity. Blood samples were collected at KKUH at approximately 12 h after the last oral dose of clozapine [14]. All blood samples were collected under steady-state conditions. These blood samples were then sent to the National Reference Laboratory in Abu Dhabi, United Arab Emirates, an outsourced laboratory service provider managed by LabCorp. The unit of the serum level measurement was nanogram/millilitre (ng/mL). Our lab reference considered levels above 650 ng/mL to be above the optimal range [38]. However, the most recent guidelines recommend clozapine levels to fall in the range of 350-600 ng/mL [39]. For patients who had more than one measurement of the serum clozapine level, only the first measurement was used in the analysis, as these patients were few in number and their doses were changed after the first measurement.

### Statistical analysis

The Statistical Package for the Social Sciences (SPSS) version 21.0 [40] was used for data analysis. Descriptive data are presented as means (M) and standard deviations (SD) or medians (Mdn) and interquartile ranges (IQR). Correlations were estimated using the bivariate correlation coefficient: the Pearson’s coefficient (*r*) for normally distributed data, and the non-parametric Spearman’s coefficient (*r*_*s*_) for non-normally distributed data. For normally distributed data, differences between groups were analysed using the Student’s t-test (*t*) or one-way analysis of variance (ANOVA) test (*F*) when appropriate. The Mann–Whitney U test (*U*) or Kruskal–Wallis one-way analysis of variance test (*H*) was used for non-normally distributed data. A hierarchical linear regression model was used to examine the predictors of serum clozapine level in our patient sample. The level of significance (*p*) was fixed at < 0.05 for analysis interpretation. The analysed dataset is available from the corresponding author upon reasonable request.

### Ethical standards

The study was approved by the Institutional Research Board Committee at the College of Medicine, King Saud University (approval reference no. 19/0054/IRB for Research Project No. E-19-4246). The requirement for informed consent was waived by the institutional review board because the study employed a retrospective observational design and patients’ data remained confidential. The authors assert that all procedures contributing to this work comply with the ethical standards of the relevant national and institutional committees on human experimentation and with the Declaration of Helsinki of 1975, as revised in 2008. The authors also followed the ICMJE guidelines in the composition of this manuscript.

## Results

The data of 94 patients who attended the Clozapine Clinic were included in the analysis. Patients’ characteristics and primary psychiatric diagnoses are presented in Table 1. The mean BMI was 29.91 kg/m^2^ (*SD* = 6.73, 95% confidence interval [CI]: 28.52–31.29), and ranging from 16.20 to 49.88 kg/m^2^, and half of the sample (48.9%) had a BMI of 30 kg/m^2^ or above. Smoking was documented in 20 (21.3%) patients, 19 of whom were men.

**Table 1:** Patients’ characteristics

	Men	Women	All patients
Median age (*IQR*)	38 (30–47)	34 (27–46)	37 (29–46)
Schizophrenia (%)	44 (46.8)	24 (25.5)	68 (72.3)
Schizoaffective, bipolar type (%)	5 (5.3)	3 (3.2)	8 (8.5)
Schizoaffective, depressive type (%)	3 (3.2)	1 (1.1)	4 (4.3)
Bipolar 1 disorder (%)	3 (3.2)	8 (8.5)	11 (11.7)
Other diagnoses (%)	2 (2.1)	1 (1.1)	3 (3.2)
Total (%):	57 (60.6)	37 (39.4)	94 (100)

*IQR* interquartile range

The prescribed dose of clozapine ranged from 75 mg to 800 mg daily, with a mean daily dose of 400 mg (*SD* = 149.69, 95% CI: 369.34–430.66). No significant difference was found in the average daily dose of clozapine between male (*M* = 408.33 mg, *SD* = 149.28) and female patients (*M* = 387.16 mg, *SD* = 151.45) (*t* (92) = 0.67, *p* = 0.506, 95% CI: − 41.78–84.12). Furthermore, no significant difference was observed in the average daily dose between patients who received additional psychotropic medications and those who received clozapine monotherapy (*p* > 0.150). The other psychotropic medications concomitantly used by patients at the time of the measurement of the serum clozapine level are presented in Table 2. In total, 64.9% of patients were taking other psychotropics in addition to clozapine.

**Table 2:** Frequency of polytherapy during clozapine treatment

Drug class	Frequency (% of total sample)
Concomitant use of drugs for depression and anxiety	38 (40.4)
Fluoxetine	9 (9.6)
Escitalopram	9 (9.6)
Fluvoxamine	4 (4.3)
Sertraline	2 (2.1)
Paroxetine	2 (2.1)
Venlafaxine	3 (3.2)
Imipramine	1 (1.1)
Clomipramine	1 (1.1)
Amitriptyline	2 (2.1)
Mirtazapine	5 (5.3)
Concomitant use of anticonvulsants or lithium	25 (25.6)
Valproate	11 (11.7)
Lamotrigine	3 (3.2)
Lithium	9 (9.6)
Lithium and valproate	1 (1.1)
Concomitant use of other drugs for psychosis	14 (14.9)
Amisulpride	3 (3.2)
Aripiprazole	9 (9.6)
Quetiapine	1 (1.1)
Risperidone	1 (1.1)

The median serum clozapine level was 705 ng/mL (*IQR* = 442.25–979.50, 95% CI: 598.51–816). The majority of patients (59.8%) had serum levels higher than 600 ng/mL, which was considered above the optimal range, as discussed above. We found that the serum clozapine level and daily dose were positively correlated (*r*_*s*_ [94] = 0.32, *p* = 0.002, 95% CI: 0.13–0.49). Table 3 shows the details of the results of the correlation analysis for all patients and the following groups: those on clozapine monotherapy, non-smokers on monotherapy, and those receiving other psychotropic medications in addition to clozapine (polytherapy). Table 3 also shows the calculated C/D ratios for these specified groups.

**Table 3:** Correlation between daily clozapine dose and serum clozapine level

Patients (N)	Serum level (ng/mL) *Mdn* ± *IQR* (Range)	Daily dose (mg) M ± SD (Range)	Correlation (*r*_*s*_) *(95% CI)*	Significance (*p*)	C/D ratio (Serum level / Daily dose)
All (94)	705 ± 442.25–979.5 (105–3388)	400 ± 149.69 (75–800)	0.321 (0.127, 0.492)	0.002	1.76
Monotherapy (33)	599 ± 262–827.5 (127–2326)	375.76 ± 150.97 (100–600)	0.396 (0.061, 0.651)	0.023	1.59
Monotherapy and Non-Smoker (22)	656.5 ± 454.75–837.25 (182–2326)	377.29 ± 160.71 (100–600)	0.480 (0.124, 0.754)	0.024	1.74
Polytherapy (61)	799 ± 505.5–1054.5 (105–3388)	413.11 ± 148.58 (75–800)	0.245 (−0.007, 0.468)	0.057	1.93

*M* mean, *SD* standard deviation, *Mdn* median, *IQR* interquartile range, *CI* confidence interval, *C/D ratio* concentration-to-dose ratio

Serum clozapine levels were significantly higher in women (*Mdn* = 843 ng/mL, *IQR* = 552–1048) than in men (*Mdn* = 607 ng/mL, *IQR* = 379–862) (*U* = 768, *p* = 0.027). Additionally, non-smokers (*Mdn* = 766 ng/mL, *IQR* = 494–985.5) had significantly higher serum clozapine levels than smokers (*Mdn* = 545 ng/mL, *IQR* = 209–843.25) (*U* = 520.5, *p* = 0.043). A trend toward a positive correlation between the serum clozapine level and BMI was noted (*r*_*s*_ [93] = 0.20, *p* = 0.051, 95% CI: − 0.01–0.39). There was no correlation between age and serum clozapine level (*r*_*s*_ [94] = − 0.06, *p* = 0.558, 95% CI -0.26, 0.14).

Patients receiving other psychotropic medications in addition to clozapine (i.e. patients on polytherapy) had significantly higher serum clozapine levels (*Mdn* = 799 ng/mL, *IQR* = 505.5–1054.5, 95% CI: 634–904) than those receiving clozapine monotherapy (*Mdn* = 599, *IQR* = 262–827.5, 95% CI: 357.64–779) (*U* = 714, *p* = 0.021). Among patients concomitantly receiving drugs for depression and anxiety (*n* = 38), those receiving fluvoxamine (*n* = 4) had the highest average serum clozapine level (*Mdn* = 2733 ng/mL, *IQR* = 2373.25–3248.75, 95% CI: 2286, 3388), which was significantly higher than the serum level in the rest of the sample (*U* = 1.00, *p* < 0.001). In addition, those receiving lamotrigine (*n* = 3) had higher average serum clozapine levels (*Mdn* = 1457 ng/mL, *IQR* = 1344.5–1810.5, 95% CI: 1232, 2164) than the rest of the sample (*U* = 19.00, *p* = 0.011). The use of any other psychotropic agent in addition to clozapine, other than fluvoxamine and lamotrigine, was not associated with a significant difference in the clozapine level (*p* > 0.05). Additional file 1 shows the C/D ratios after excluding fluvoxamine and lamotrigine.

The median norclozapine level was 301 ng/mL (*IQR* = 220–482.25, 95% CI: 277.5, 355.99). The calculated serum CLZ/NDMC ratio ranged from 1 to 8 (*Mdn* = 2.14, *IQR* = 1.66–2.47, 95% CI; 1.91, 2.22). The serum CLZ/NDMC ratio was positively correlated with the serum clozapine level (*r*_*s*_ [94] = 0.357, *p* < 0.001, 95% CI: 0.166, 0.522). No patient had a ratio lower than 0.5, suggesting no recent nonadherence to clozapine. However, 13 patients (13.8%) had ratios higher than 3, indicating metabolizing enzyme saturation or inhibition. All patients on fluvoxamine (*n* = 4) had ratios higher than 3.

Hierarchical multiple linear regression was performed to explore which variables predicted significant variation in the serum clozapine level. The predictor variables were selected based on the previous literature and the present analyses (i.e. variables showing a significant difference in serum clozapine level in our patients). These predictor variables included the following clinical variables: daily clozapine dose, sex, smoking status, BMI, and concomitant use of fluvoxamine and/or lamotrigine. The model employed a log transformation of serum clozapine level values as the dependent variable (to handle skewed data), as in previous predictive models of the serum clozapine level [28, 31]. Sex as a predictor was removed, as female sex was collinear with increased BMI (*p* = 0.004) and non-smoker status (*p* < 0.001). In addition, the hierarchical procedure demonstrated that the addition of sex as a predictor did not contribute significantly to the log-transformed serum clozapine level (*p* = 0.87). The resultant model explained a significant amount of variance (43.6%) in the serum clozapine level (Table 4). The use of fluvoxamine was the most important predictor of increased serum clozapine levels and predicted an increase of approximately 455.9%. The second most important predictor was daily dose; each 1-mg increase in dose predicted a 0.23% increase in the serum clozapine level. In addition, non-smoker status predicted a 51.4% increase in the serum clozapine level, whereas the use of lamotrigine predicted a significant increase in the serum clozapine level of approximately 112.8%. The last predictor in our model was BMI, with each 1-kg/m^2^ increment in BMI predicting a 1.86% increase in the serum clozapine level. The model equation for predicting the serum clozapine level in our sample of Middle Eastern patients was as follows: log_10_ (clozapine level) = 2.135 + (0.745 × fluvoxamine use) + (0.001 × clozapine dose) + (0.180 × being a non-smoker) + (0.328 × lamotrigine use) + (0.008 × BMI), with categorical clinical variables represented by 1 if present.

**Table 4:** Multiple linear regression of the serum clozapine level (*N* = 94)

Clinical predictor	B	95% CI	***beta***	***t***	***p***
(Constant)	2.135	(1.879–2.391)		16.57	< 0.001
Use of fluvoxamine	0.745	(0.516, 0.975)	0.511	6.46	< 0.001
Clozapine daily dose	0.001	(0.001, 0.001)	0.323	4.08	< 0.001
Non-smoker	0.180	(0.066, 0.293)	0.249	3.14	0.002
Use of lamotrigine	0.328	(0.064, 0.592)	0.196	2.47	0.016
Body mass index	0.008	(0.001, 0.015)	0.177	2.22	0.029

Dependent variable: log_10_ (serum clozapine level)

Model fit: *F* (6, 86) = 15.246, *p* < 0.001, R^2^ = 0.467, R^2^_Adjusted_ = 0.436

*CI* confidence interval

## Discussion

To the best of our knowledge, the present study is the largest study to date to investigate serum clozapine levels in Middle Eastern patients; moreover, it is the first study on this research topic in more than two decades [36]. Similar to previous studies, the present study demonstrated a linear relationship between the daily clozapine dose and its serum level [18, 30, 35]. However, this linearity is an approximation since higher levels of clozapine can lead to enzymatic saturation, which causes small dose increases to result in significant increases in the serum level, producing a non-linear relationship [41].

The present study results are also consistent with our clinical observation that the association between clozapine dose and serum level in patients with Middle Eastern ethnicity resembles that found in East Asians as opposed to European Caucasians [18, 25, 32–34, 37]. In our sample, the C/D ratio calculated from the average clozapine level and daily dose was 1.59 for patients on clozapine monotherapy, but varied by sex and smoking status (Table 3 and Additional file 1). This C/D ratio is closer to that found in Asians and Native Americans than in Europeans [23]. The average serum clozapine level in our patients was markedly above the recommended level for clinical response [24, 39], which might be attributable to the lack of clozapine TDM prior to the establishment of the clozapine clinic and the reliance of clinicians on dosing regimens based on studies on patients of European descent [42].

Moreover, in our sample, female patients had significantly higher serum clozapine levels than male patients, as observed in other patient samples [28, 31, 35]. However, our correlational analysis suggested that the factor of female sex was confounded by smoking status and BMI. We observed that an increase in BMI predicted increased serum clozapine level, which is consistent with a previous study suggesting a positive correlation between weight and serum clozapine level [31]. Since approximately half of our patients were obese, this may partly explain the high clozapine levels in our sample. Smoking status was strongly associated with clozapine level in our patients, which is consistent with numerous reports demonstrating that smoking cigarettes is associated with decreased serum clozapine level [29, 31, 43]. However, this association was only observed in our male patients, as there was an inadequate number of female smokers in our sample for statistical analysis.

Our results demonstrated that a higher serum CLZ/NDMC ratio was significantly associated with a higher serum clozapine level. This association was previously demonstrated in a study of more than 100,000 clozapine TDM samples [25]. Monitoring this ratio may have another clinical utility, since there is a relationship between increased CLZ/NDMC ratio and fewer metabolic side effects of clozapine [44]. We also observed that ratios above 3 were found in patients taking fluvoxamine, a known CYP1A2 inhibitor, thus supporting the finding that the CLZ/NDMC ratio is a good indicator of CYP1A2 enzyme activity [15]. It is well established that the use of fluvoxamine increases the serum clozapine level significantly [45], and in our patients, the use of fluvoxamine was the most significant predictor of higher serum clozapine level. Furthermore, we observed that the use of lamotrigine also predicted a significant increase in clozapine serum level. However, this effect has only been documented in one case report [46], and very few patients were receiving lamotrigine in our sample. Other researchers have concluded that lamotrigine does not significantly affect clozapine metabolism [47] or may actually be a mild inducer of its metabolism [48].

Using our predictive model (detailed in Table 4), we calculated that Middle Eastern patients with a BMI of 25 kg/m^2^ required a dose between 50 mg/daily and 275 mg/daily for non-smokers, and a dose between 200 mg/daily and 450 mg/daily for smokers to reach a predicted serum clozapine level between 350 to 600 ng/mL. Patients with higher BMIs would require lower doses to avoid reaching levels above the optimal range. Maximum doses could not be calculated for patients concomitantly receiving fluvoxamine or lamotrigine because the model predicted levels above the optimal range (higher than 600 ng/mL) for such patients, especially if they were non-smokers. It is important to note that these findings are not a substitute for TDM in clinical practice due to the small sample size in the present study and because these calculated dose values are based on approximate definitions of the optimal range of clozapine serum level.

Since the association between serum clozapine level and clinical response is believed to be curvilinear [49], clinicians treating patients of Middle Eastern descent are encouraged to utilise TDM in clozapine therapy to ensure treatment success. If TDM is unavailable, our predictive model may serve as a preliminary guide to mitigate the reluctance of clinicians to prescribe clozapine to Middle Eastern patients. Our findings highlight both the benefits of studying psychotropic medications in different ethnic groups and the risk of neglecting ethnic differences in biomedical research at large [50]. Because of these benefits and risks, there have been increasing efforts to explore the clozapine C/D ratio in non-European populations, which have shown results similar to the present study [51, 52].

This study has some limitations, including its small sample size, retrospective design, and reliance on clinical notes in electronic medical records. The collected data did not include possible confounding factors such as dietary habits, caffeine intake, non-psychotropic medication use, and inflammatory states, which may affect drug metabolism. In our analysis, we relied on a single measure of the serum clozapine level, which cannot account for any intra-individual variation in clozapine kinetics. In determining our sample of patients as Middle Eastern, we relied on nationality and family or tribal affiliations, but did not conduct any testing to confirm ethnicity. Moreover, we could not collect data on possible deviation from the standardised time of sampling by the laboratory personnel. Since our sample was composed of outpatients, the data may be contaminated by a lack of adherence. In addition, our sample was heterogeneous in terms of diagnoses and treatment regimens. Additionally, to generate our predictive model of the serum clozapine level, we used multiple linear regression, which can have issues with collinearity since there was only a single female smoker in our sample. Finally, the average serum clozapine level in our patient sample reflected current practice, but is not endorsed by the authors.

## Conclusions

The present study provides a naturalistic examination of clozapine TDM, reflecting daily practice in the Middle Eastern country of Saudi Arabia. The findings demonstrate a relationship between clozapine dose and serum level in Middle Eastern patients, which when compared to previous reports, suggest that clozapine C/D ratios in Middle Easterners are more similar to Asians than to Europeans. In addition, we provide a predictive model to estimate the serum clozapine level using common variables, which may be clinically useful, notwithstanding its limitations. The model demonstrates how patients receiving other psychotropics, especially fluvoxamine, and patients with comorbid obesity are significantly more likely to have a high serum clozapine level. These findings may be helpful to clinicians prescribing clozapine to Middle Easterners, but should not be considered a substitute to individualised TDM. Future studies should investigate the association between clozapine dose and serum level in larger samples of Middle Eastern patients. In order to better establish clozapine C/D ratios in patients of Middle Eastern ethnicity, studies that analyse repeated samples of patients on clozapine, while eliminating major confounders, are needed. Lastly, more studies are needed to further explore the association between serum clozapine level and clinical response in this demographic.

## Supplementary Information

**Additional file 1.**

## Data Availability

The datasets generated and/or analysed during the current study are not publicly available due to confidentiality considerations, but are available from the corresponding author on reasonable request.

## Abbreviations

BMI: Body mass index
C/D ratio: Concentration-to-dose ratio
CI: Confidence interval
CLZ/NDMC: Clozapine to norclozapine level
CYP: Cytochrome
IQR: Interquartile range
KKUH: King Khalid University Hospital
M: Mean
Mdn: Median
SD: Standard deviation
TDM: Therapeutic drug monitoring
