# Supplementary material for: Factors predicting serum clozapine levels in Middle Eastern patients: an observational study
Source: BMC Psychiatry. 2022 Apr 15;22:269. doi: 10.1186/s12888-022-03910-6 (PMC9011948; doi:10.1186/s12888-022-03910-6)
Supplement: Supplementary file 1 — Additional file 1. [file 12888_2022_3910_MOESM1_ESM.docx]

| Patients (N) | Serum level (ng/mL)  *Mdn* ± *IQR*  (Range) | Daily dose (mg)  M ± SD  (Range) | C/D ratio ( Serum level / Daily dose) |
| --- | --- | --- | --- |
| Female Non-smoker^*^ (34) | 842.5 ± 539.25–999  (187–2326) | 384.56 ± 156.79  (75–800) | 2.19 |
| Male Non-smoker^*^ (34) | 594.5 ± 425.75–817.75  (182–1455) | 406.62 ± 152.05  (100–600) | 1.46 |
| Male Smoker^*^ (18) | 479.5 ± 188.5–787.75  (105–1284) | 416.67 ± 159.96  (100–700) | 1.15 |
| ^*^Excluding patients on fluvoxamine or lamotrigine.  M: mean; SD: standard deviation; Mdn: median; IQR: interquartile range; CI: confidence interval; C/D ratio: concentration-to-dose ratio | | | |

**Additional file 1. Clozapine C/D ratios stratified by sex and smoking status:**
